# Normal size of benign upper neck nodes on MRI: parotid, submandibular, occipital, facial, retroauricular and level IIb nodal groups

**DOI:** 10.1186/s40644-022-00504-z

**Published:** 2022-12-08

**Authors:** Qi Yong H. Ai, Tiffany Y. So, Kuo Feng Hung, Ann D. King

**Affiliations:** 1 Department of Health Technology and Informatics, The Hong Kong Polytechnic University, Hong Kong S.A.R, P.R. China; 2 Department of Imaging and Interventional Radiology, Faculty of Medicine, The Chinese University of Hong Kong, Prince of Wales Hospital, Hong Kong S.A.R, P.R. China; 3 Division of Oral and Maxillofacial Surgery, Faculty of Dentistry, University of Hong Kong, Hong Kong S.A.R, P.R. China

**Keywords:** Normal nodal size, MRI, head and neck, Benign and reactive, Lymph nodes

## Abstract

**Purpose:**

Nodal size is an important imaging criterion for differentiating benign from malignant nodes in the head and neck cancer staging. This study evaluated the size of normal nodes in less well-documented nodal groups in the upper head and neck on magnetic resonance imaging (MRI).

**Methods:**

Analysis was performed on 289 upper head and neck MRIs of patients without head and neck cancer. The short axial diameters (SAD) of the largest node in the parotid, submandibular, occipital, facial, retroauricular and Level IIb of the upper internal jugular nodal groups were documented and compared to the commonly used threshold of ≥ 10 mm for diagnosis of a malignant node.

**Results:**

Normal nodes in the parotid, occipital, retroauricular and Level IIb groups were small with a mean SAD ranging from 3.8 to 4.4 mm, nodes in the submandibular group were larger with a mean SAD of 5.5 mm and facial nodes were not identified. A size ≥ 10 mm was found in 0.8% of submandibular nodes. Less than 10% of the other nodal group had a SAD of ≥ 6 mm and none of them had a SAD ≥ 8 mm.

**Conclusion:**

To identify malignant neck nodes in these groups there is scope to reduce the size threshold of ≥ 10 mm to improve sensitivity without substantial loss of specificity.

**Supplementary Information:**

The online version contains supplementary material available at 10.1186/s40644-022-00504-z.

## Background

The spread of head and neck cancer to metastatic nodes in the neck has an important impact on management and prognosis [1–5]. Imaging is used to detect the presence of metastatic nodes and map the extent of disease from the first echelons of nodal spread to subsequent nodal groups down the neck. This information is crucial for neck dissection and radiotherapy field planning and for those cancers treated non-surgically, imaging is the only method to stage the disease [2, 5–7]. The diagnosis of a metastatic node on cross-sectional imaging is dependent on meeting imaging criteria that discriminate malignant nodes from the normal or reactive nodes commonly seen on images of the head and neck [8, 9].

The three main morphological criteria for a malignant node on magnetic resonance imaging (MRI) are size, necrosis and extranodal spread [8, 9]. Of these, the most common feature of a metastatic node is large size, with necrosis and extranodal spread being less commonly encountered [10–12] and therefore of most value in non-enlarged nodes. As malignant nodes tend to be round and reactive nodes oval in shape, the short axis diameter (SAD) on axial images is frequently the measurement of choice [8, 13–15]. The size threshold chosen to detect a malignant node is of course always a trade-off between sensitivity and specificity [8, 16–20] and may vary between centers and may be influenced by the clinical scenario [21]. However, commonly used thresholds in practice and research are SAD thresholds ≥ 11 mm for the jugulodigastric nodes and ≥ 10 mm for other nodes in the head and neck [8, 22, 23]. The exception is the retropharyngeal nodes where a lower threshold of ≥ 5 or 6 mm is used to reflect the smaller size of normal and reactive nodes in this group [9, 13, 20]. However, benign nodes in the parotid, submandibular, occipital groups and Level IIb of the upper internal jugular group (posterior and separate to the vein) are also usually much smaller than nodes in Level IIa of the upper internal jugular group (adjacent to the vein), but the normal size range of these nodal groups is poorly documented in the literature. Furthermore, nodes in facial and retroauricular groups are rarely observed. Using the threshold of 10 mm in these groups could reduce sensitivity for metastatic nodal spread from head and neck cancers that drain to these sites. The lower frequency of metastatic nodes in these groups, compared to those along the internal jugular chain, make it difficult to obtain sufficient data from surgical series to define optimum size thresholds to divide malignant from benign nodes. A study of nodal size in patients without head and neck cancer should at least shed light on the expected upper limit of size of normal/reactive benign nodes.

In this study we documented the expected range of size for benign nodes in the parotid, submandibular, occipital, Level IIb, facial and retroauricular groups by measuring the SAD of the largest nodes in these groups on the upper neck MRI images of patients without cancer. We also measured the SAD of the already well-documented nodes in the upper internal jugular (jugulodigastric and other Level IIa) and retropharyngeal groups, to compare the size of these nodes in our cohort with those previously reported in the literature. Finally, for each nodal group we documented the frequency of nodes, correlated the size of the largest node with age and measured nodal sizes in both sides of the neck to determine the expected discrepancy between the right and the left sides of the neck.

## Methods

### Patients

This retrospective study was performed with approval from the local institutional review board and written informed consent was waived. This study analysed 289 upper head and neck MRI scans of patients who were referred to our institution between 2005 and 2016 for suspected NPC due to the raised plasma Epstein-Barr Virus (EBV)-DNA but who had no head and neck cancer diagnosed at a minimum follow-up of 2 years. The median age of the included patients was 53 years (range: 28 -78 years).

### MRI acquisition

The upper head and neck MRI was performed using a 1.5 T or 3 T whole-body MRI system (Philips Healthcare, Best, the Netherlands) and comprised of at least (1) axial fat-suppressed T2-weighted (repetition time/ echo time, 2500—4000/ 80—100 ms; field of view, 22 cm; section thickness, 4 mm without a slice gap; number of slices, 30; echo train length, 15–17; sensitivity encoding factor, 1; number of signal acquired, 2), (2) axial T1-weighted images (repetition time/ echo time, 500/ 10—20 ms; field of view, 22 cm; section thickness, 4 mm without a slice gap; number of slices, 30; echo train length, 4; sensitivity encoding factor, 1; number of signal acquired, 2) with or without (3) coronal T2- or T1-weighted images, and (4) contrast-enhanced axial T1-weighted images.

### MRI analysis

**Fig. 1:**
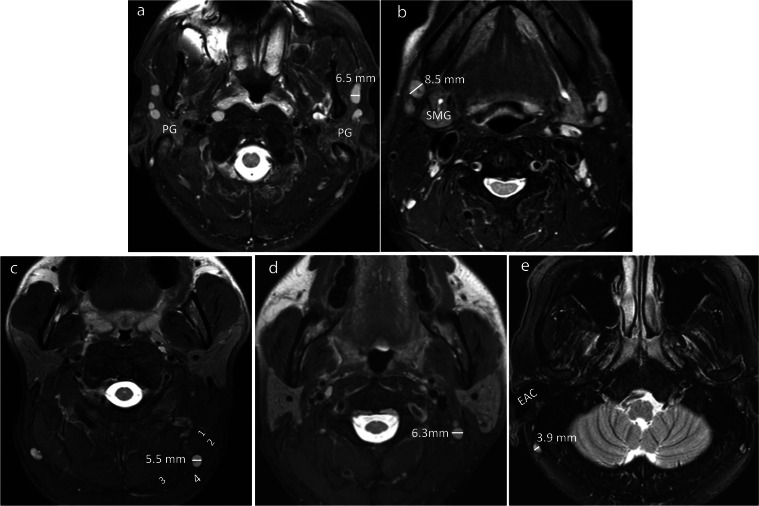
Axial T2-weighted fat-suppressed MR images of five patients without head and neck cancer with normal/benign reactive nodes in the less documented nodal groups in the upper head and neck, comprising parotid (**a**), submandibular (**b**), occipital (**c**), Level IIb (**d**) and retroauricular nodes (**e**). The mean SADs of the largest nodes for parotid, submandibular, occipital, Level IIb, and retroauricular nodes were 4.4 ± 1.0 mm, 5.5 ± 1.4 mm, 3.8 ± 1.0 mm, 4.3 ± 1.2 mm, and 3.8 ± 0.7 mm, respectively. (PG = parotid gland, SMG = submandibular gland, EAC = external auditory canal, SAD = short axis diameter, 1 = Splenius capitis muscle, 2 = sternocleidomastoid muscle, 3 = trapezius muscle, 4 = occipital subcutaneous fat)

**Fig. 2:**
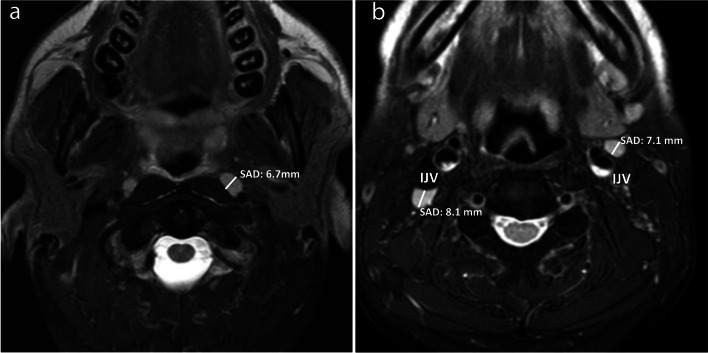
Axial T2-weighted fat-suppressed MR images of two patients without head and neck cancer showing bilateral normal/benign reactive retropharyngeal (**a**), left jugulodigastric (**b**) and right Level IIa (**b**) nodes. The mean SADs of the largest nodes for retropharyngeal, jugulodigastric, and Level IIa nodes were 3.9 ± 1.2 mm, 7.4 ± 1.9 mm, and 4.9 ± 1.6 mm, respectively. (IJV = internal jugular vein, SAD = short axis diameter)

Nodal groups based on sites described by Som et al. and Gregoire et al. [22–24] were examined, comprising six less documented groups which were (1) parotid, (2) submandibular, (3) occipital, (4) Level IIb, (5) facial and (6) retroauricular (Fig. 1a-e), together with (7) retropharyngeal, (8) jugulodigastric region and (9) Level IIa (other than the jugulodigastric node) (Fig. 2a-b). The SAD of the largest node in each group was measured on the MRI images in the axial plane. Only nodes ≥ 2.0 mm in SAD were considered to be measurable and included in the study. Nodal size was documented using the SAD by a researcher with 7 years experiences in head and neck imaging. Measurements were also performed for inter-observer assessment on 50 randomly selected MRIs by a radiologist with 3 years of experiences in head and neck radiology.

### Statistical analysis

For each nodal group, frequency of nodes was based on patients who had a node in either side of the neck and the SAD was based on the size of the largest node. For patients with bilateral nodes, the paired t-test (data normally distributed) or Wilcoxon signed-rank test (data non-normally distributed) compared the differences in the SAD of the largest node in each nodal group between the right and left sides of the neck. For patients with unilateral nodes, the student t-test (data normally distributed) or Mann–Whitney test (data non-normally distributed) compared differences in SAD of the largest node in each group with the size of the largest node in those with bilateral nodes. The normality distribution of data was evaluated using Kolmogorov–Smirnov test. The SAD of the largest node in each nodal group was correlated with age using the Pearson correlation test and the Pearson coefficient was calculated. All statistical tests were 2-sided, and a *p*-value < 0.05 was considered to indicate a statistically significant difference. Analyses were performed using the statistical analysis software SPSS (version 26.0; IBM). Inter-observer agreement for the SAD of the largest node in each nodal group were evaluated using the intra-class correlation test and the intra-class coefficient (ICC) was calculated.

## Results

### Frequency of patients with the nodes in each nodal group

The frequencies of nodes in descending order were the jugulodigastric (98.2%) > submandibular (90.3%) > Level IIa (81.6%) > Level IIb (79.2%) > parotid (74.4%) > retropharyngeal (49.1%) > occipital (27.7%) > retroauricular (2.8%) (Table 1). Nodes were not identified in facial group (Table 1). For the parotid group, 80.0% (172/215) of the largest nodes were in the superficial lobe of which 68.0% (117/172) were at a constant location in the anterior portion (Fig. 1a), 20.0% (43/215) were in the deep lobe.

**Table 1:** Frequency and SAD of the largest node in each nodal group

Nodal groups	Frequency of patients with nodes	SAD^a^ (mm)	Number (percentage) of nodes with a SAD > current size criteria for a malignant node^b^
Parotid	215 (74.4%)	4.4 ± 1.0 (2.2 – 7.3)	0 (0%)
Submandibular	261 (90.3%)	5.5 ± 1.4 (2.7 – 10.8)	2 (0.8%)
Occipital	80 (27.7%)	3.8 ± 1.0 (2.1 – 6.1)	0 (0%)
Facial	0 (0%)	0 (-)	0 (0%)
Retroauricular	8 (2.8%)	3.8 ± 0.7 (3.3 – 4.8)	0 (0%)
Retropharyngeal	142 (49.1%)	3.9 ± 1.2 (2.1 – 9.0)	22 (15.5%)
Upper internal jugular			
Jugulodigastric	284 (98.2%)	7.4 ± 1.9 (2.8 – 12.6)	15 (5.3%)
Level IIa	236 (81.6%)	4.9 ± 1.6 (2.3 – 11.9)	3 (1.3%)
Level IIb	229 (79.2%)	4.3 ± 1.2 (2.1– 7.6)	0 (0%)

*SAD* Short axis diameter

^a^data shown as mean value ± standard deviation (range)

^b^SAD of ≥ 5 mm, 11 mm, and 10 mm for identifying malignant retropharyngeal nodes, jugulodigastric nodes, and any other head and neck nodes respectively

### Size of the largest nodes in each nodal group

The SADs of the largest nodes in descending order were jugulodigastric (7.4 ± 1.9 mm) > submandibular (5.5 ± 1.4 mm) > Level IIa (4.9 ± 1.6 mm) > parotid (4.4 ± 1.0 mm) > Level IIb (4.3 ± 1.2 mm) > retropharyngeal nodes (3.9 ± 1.2 mm) > occipital nodes (3.8 ± 1.0 mm) and retroauricular nodes (3.8 ± 0.7 mm) (Table 1). The range of size of nodes in each group is shown in Supplementary Table 1. The percentage of benign nodes larger than the current thresholds for a malignant node were 15.5% (22/142) for retropharyngeal, 5.3% (15/284) for jugulodigastric, 1.3% (3/236) for Level IIa and 0.8% (2/261) for submandibular groups (Table 1). Less than 10% of nodes in the parotid, occipital, Level IIb, retropharyngeal and retroauricular groups had a SAD of ≥ 6 mm and none of the nodes in the parotid, occipital, Level IIb, and retroauricular groups had a SAD ≥ 8 mm (Supplementary Table 1).

### Comparison of size of nodes in each side of the neck

The SAD of the largest node in each group was significantly smaller when nodes were unilateral compared to bilateral (*p* < 0.01 to 0.048, Table 2) but for bilateral nodes there was no significant difference between the right and left sides (*p* = 0.14 to 0.92) (Table 3).

**Table 2:** Frequency and SAD of the largest node in patients with unilateral and bilateral nodes

Nodal groups	Number of patients with unilateral nodes	Number of patients with bilateral nodes	SAD of the largest node		
			Unilateral*	Bilateral*	*P*-value
Parotid	81 (28.0%)	134 (46.4%)	4.1 ± 1.0	4.6 ± 0.9	< 0.01
Submandibular	57 (19.7%)	204 (70.6%)	4.7 ± 1.2	5.7 ± 1.4	< 0.01
Occipital	58 (20.1%)	22 (7.6%)	3.6 ± 0.9	4.1 ± 1.0	0.048
Retroauricular	8 (2.8%)	0 (0%)	3.8 ± 0.7	-	-
Retropharyngeal	59 (20.4%)	83 (28.7%)	3.5 ± 0.9	4.2 ± 1.3	< 0.01
Upper internal jugular					
Jugulodigastric	20 (6.9%)	264 (91.3%)	6.1 ± 1.9	7.5 ± 1.9	< 0.01
Level IIa	79 (27.3%)	157 (54.3%)	4.2 ± 1.1	5.3 ± 1.6	< 0.01
Level IIb	56 (19.4%)	173 (59.8%)	3.7 ± 1.1	4.5 ± 1.1	< 0.01

All data groups are normally distributed (*p* > 0.05 using one sample Kolmogorov–Smirnov test)

*SAD* Short axis diameter

^*^data shown as mean value ± standard deviation

**Table 3:** The SAD of the largest node in the left and right side in patients with bilateral nodes in the same group^a^

Nodal groups	SAD in patients with bilateral nodes		
	Left	Right	*P*-value
Parotid (*n* = 134)	4.2 ± 0.9	4.1 ± 1.0	0.19
Submandibular (*n* = 204)	5.0 ± 1.5	5.0 ± 1.5	0.92
Occipital (*n* = 22)	3.8 ± 1.1	3.9 ± 1.2	0.71
Retropharyngeal (*n* = 83)	3.9 ± 1.1	3.7 ± 1.2	0.14
Upper internal jugular			
Jugulodigastric (*n* = 264)	6.7 ± 2.0	6.8 ± 1.9	0.36
Level IIa (*n* = 157)	4.7 ± 1.6	4.5 ± 1.6	0.14
Level IIb (*n* = 173)	4.0 ± 1.2	4.0 ± 1.1	0.86

All data groups are normally distributed (*p* > 0.05 using one sample Kolmogorov–Smirnov test)

*SAD* short axis diameter

^a^No patients with facial or retroauricular nodes had bilateral nodes

### Correlation of nodal size in each group with age

Age negatively correlated with the SAD of the largest node in the retropharyngeal and upper internal jugular group (jugulodigastric nodes, Level IIa, and Level IIb nodes) (Pearson correlation coefficients: -0.19 to -0.15, *p* < 0.01 to 0.02) (Supplementary Fig. 1), but not with parotid, submandibular, occipital and retroauricular nodes (*p* = 0.09 to 0.57) (Table 4).

**Table 4:** Correlation of age with SAD of the largest nodes in each nodal group^a^

Nodal groups	SAD^b^ (mm)	Pearson Coefficient	*P*-value
Parotid	4.4 ± 1.0 (2.2 – 7.3)	-0.12	0.09
Submandibular	5.5 ± 1.4 (2.7 – 10.8)	-0.10	0.12
Occipital	3.8 ± 1.0 (2.1 – 6.1)	-0.11	0.57
Retroauricular	3.8 ± 0.7 (3.3 – 4.8)	-0.12	0.13
Retropharyngeal	3.9 ± 1.2 (2.1 – 9.0)	-0.19	**0.02**
Upper internal jugular			
Jugulodigastric	7.4 ± 1.9 (2.8 – 12.6)	-0.18	** < 0.01**
Level IIa	4.9 ± 1.6 (2.3 – 11.9)	-0.15	**0.02**
Level IIb	4.3 ± 1.2 (2.1– 7.6)	-0.16	**0.01**

*SAD* Short axis diameter

^a^No patient had facial nodes

^b^data shown as mean value ± standard deviation (range)

### Inter-observer agreement

Inter-observer agreement for SAD of the largest nodes in the nodal groups showed ICCs ranged from 0.82 to 0.93 (all *p* < 0.01) (Supplementary Table 2).

## Discussion

This study documented the frequency and range in size of normal/reactive benign nodes in nodal groups of head and neck that have received less scrutiny in the literature, namely the parotid, submandibular, occipital, Level IIb nodes, facial and retroauricular groups. Measurable nodes (i.e., those ≥ 2 mm) were most frequent in the submandibular (90.3%), followed by Level IIb (79.2%), parotid (74.4%), occipital (27.7%) and retroauricular (2.8%) groups, and no nodes were identified in the facial group. Nodes in the parotid, occipital, Level IIb, and retroauricular groups were small ranging in mean SAD from 3.8 to 4.4 mm, while nodes in the submandibular group were slightly larger with a mean SAD of 5.5 mm. The range of nodal size in each of these groups only surpassed the threshold of ≥ 10 mm for a malignant node in 0.8% of submandibular nodes and none of the nodes in the other groups.

Nodal sizes for retropharyngeal, jugulodigastric and other Level IIa nodes were in keeping with sizes reported in the literature, suggesting our group is fairly representative of the expected size of normal/reactive nodes [8, 9, 13, 25, 26]. Of note, the largest node in the neck was the jugulodigastric node followed by the other Level IIa nodes. The larger size of jugulodigastric, Level IIa and submandibular nodes may reflect stimulation by dental disease or upper respiratory tract infections. The size of the retropharyngeal nodes was similar to nodes in the parotid, Level IIb, occipital groups and retroauricular groups. The smaller nodal size in the upper internal jugular chain in Level IIb compared to Level IIa, is of special interest in these patients being screened for NPC, because Level IIb nodes are commonly the first echelon of nodal spread in NPC [27]. The results in this study suggest that applying the current threshold of ≥ 10 mm to parotid, occipital, Level IIb nodes, facial and retroauricular nodes would result in a very high specificity for a malignant node but would compromise sensitivity. Recently Elsholtz et al. [28], proposed using a threshold of < 5 mm to denote normal nodes in the facial, parotid, retroauricular, occipital groups for a Node-RADS system that categorises the likelihood of a metastatic node from 1 to 5. Our findings support lowering the threshold from 10 mm to one that is similar to that already applied to retropharyngeal nodes, i.e., 5 mm or 6 mm [9, 13, 20]. Our current results suggest that 6 mm, rather than 5 mm, would reduce the number of false positive nodes and improve specificity in the parotid, occipital, Level IIb nodes, as well as the retropharyngeal group. However, thresholds may need to be adjusted 1- 2 mm higher or lower for submandibular and retroauricular groups respectively and the presence of any node in the facial group. Moreover, size thresholds are a trade-off between sensitivity and specificity and chosen thresholds may need to be adapted to the clinical scenario; for example to improve sensitivity when searching for small metastases in surgical candidates with a clinically N0 neck, thresholds as low as ≥ 4 mm [17, 29, 30] may be used for ultrasound guided fine needle aspiratory cytology of submandibular and upper internal jugular nodes.

Unilateral nodes or nodes larger on one side than the other is often used as a sign to heighten suspicion of a metastatic node in groups along the expected pathway of nodal spread. The results of this study support this practice for unilateral nodes, especially in the jugulodigastric region where only 6.9% were unilateral, compared to 19–28% in the other nodal groups. Interestingly, when nodes were bilateral, they were larger than when unilateral which may represent a general stimulation of nodes, in our group this may possibly be due to EBV infection causing reactive nodes. However, once nodes were bilateral nodes there was no significant difference in size between the right and left sides of the neck, again suggesting that asymmetry in size should be regarded with suspicion.

This study found age negatively correlated with the SAD of nodes in retropharyngeal group, and upper jugular group (jugulodigastric nodes, Level IIa and Level IIb nodes) as shown in previous studies [25, 31]. However, we found no correlation between age and size of nodes in the parotid, submandibular and occipital groups.

Although this study has focused on size criterion, it is worth remembering that the imaging diagnosis of a metastatic node, especially for those small nodes that do not surpass the size threshold, also takes account of other morphological features such as shape, necrosis, heterogeneity, extranodal spread, hilum, vascular pattern and functional features such as F-fluorodeoxyglucose activity and restricted diffusion.

There are some limitations in this study. First, evaluation of nodal groups was limited to those groups consistently covered in all scans of the upper neck, which unfortunately did not include submental nodes. Second, this study only evaluated measurable nodes (a SAD of ≥ 2 mm) which may result in under reporting of the frequency of the nodes in each nodal group. However, this assured the certainty in identifying a node rather than other structures (i.e., small vessels). Third, all patients in this study were from a single institution but this group should represent the expected range in size of benign nodes (normal and reactive). Although this group of patients with persistently elevated plasma EBV-DNA may have a potential bias towards reactive, and hence larger size, benign nodes, this adds to strength to the findings that a reduction in nodal size threshold for detecting malignant nodes should not compromise specificity. Fourth, the influence of outliers on the statistical significance between age and SAD is unknown. Fifth, the diagnostic performance of the SAD thresholds could not be fully assessed because we only evaluated the SAD of benign and not metastatic nodes. Radiology studies that include both malignant and benign nodes with pathological correlation are needed to explore the diagnostic performance of size thresholds for these under reported nodal groups, and this may require multicenter studies as data from a single center may be insufficient for analysis.

## Conclusion

This study examined the frequency and size of benign nodes in the parotid, submandibular, occipital, level IIb, facial and retroauricular nodes in the upper neck. Most patients had nodes in each of these groups on imaging, with the exception of the facial group. Nodes in these groups were small (mean SAD ranging from 3.8 to 4.4 mm) while nodes in the submandibular group were slightly larger (mean SAD of 5.5 mm).

Reducing the size threshold of nodes in the head and neck is known to improve sensitivity for identifying malignant nodes in the head and neck and our results suggest this may be possible without substantial loss of specificity in these less well documented nodal groups. Our data should encourage future investigation of sensitivity and specificity using a lower threshold for detection of malignant nodes (i.e., ≥ 6–7 mm for parotid, occipital and Level IIb groups or 1–2 mm higher for submandibular group or lower for retroauricular group). Results also support heighten suspicion of metastatic nodes when nodes in groups along the expected pathway of nodal spread are unilateral or larger on one side than the other.

## Supplementary Information

**Additional file 1:** **Supplementary Table 1.** Frequency of the nodes according to range in SAD of the largest node*. **Supplementary Table 2.** Inter-observer agreement for SAD of the largest nodes*. **Supplementary Figure 1.** Scatter plots show the negative correlation of age with the short axis diameter of the largest node inretropharyngeal (a), jugulodigastric (b), Level IIa (c) and Level IIb (d) nodes. The Pearson correlation coefficients are -0.19, -0.18, -0.15, and -0.16 for retropharyngeal, jugulodigastric, Level IIa and Level IIb nodes,respectively.

## Data Availability

The datasets of current study are available from the corresponding author on reasonable request.

## Abbreviations

MRI: Magnetic resonance imaging
SAD: Short axis diameter
NPC: Nasopharyngeal carcinoma
EBV: Epstein-Barr virus
DNA: Deoxyribonucleic acid
ICC: Intra-class coefficient
PG: Parotid gland
SMG: Submandibular gland
IJV: Internal jugular vein
