# Supplementary material for: Normal size of benign upper neck nodes on MRI: parotid, submandibular, occipital, facial, retroauricular and level IIb nodal groups
Source: Cancer Imaging. 2022 Dec 8;22:66. doi: 10.1186/s40644-022-00504-z (PMC9730594; doi:10.1186/s40644-022-00504-z)
Supplement: Supplementary file 1 — Additional file 1: Supplementary Table 1. Frequency of the nodes according to range in SAD of the largest node*. Supplementary Table 2. Inter-observer agreement for SAD of the largest nodes*. Supplementary Figure 1. Scatter plots show the negative correlation of age with the short axis diameter of the largest node inretropharyngeal (a), jugulodigastric (b), Level IIa (c) and Level IIb (d) nodes. The Pearson correlation coefficients are -0.19, -0.18, -0.15, and -0.16 for retropharyngeal, jugulodigastric, Level IIa and Level IIb nodes,respectively. [file 40644_2022_504_MOESM1_ESM.docx]

**Supplementary files**

Supplementary Table 1. Frequency of the nodes according to range in SAD of the largest node*

| Short axis diameter | Parotid nodes  (n = 215) | Submandibular nodes  (n =261) | Occipital nodes  (n = 80) | Retroauricular  Nodes  (n =8) | Retropharyngeal nodes  (n = 142) | Jugulodigastric nodes  (n = 284) | Level IIa nodes  (n =236) | Level IIb nodes  (n = 229) |
| --- | --- | --- | --- | --- | --- | --- | --- | --- |
| < 3.0mm | 13  (6.0%) | 2  (0.8%) | 20  (25.0%) | 0  (0%) | 26  (18.3%) | 1  (0.4%) | 19  (8.2%) | 20  (8.7%) |
| ≥ 3.0mm,  < 5.0mm | 138  (64.3%) | 99  (37.9%) | 51  (63.8%) | 8  (100%) | 94  (66.2%) | 26  (9.2%) | 117  (49.6%) | 155  (67.7%) |
| ≥ 5.0mm,  < 6.0mm | 49  (22.8%) | 77  (29.5%) | 7  (8.8%) | 0  (0%) | 15  (10.6%) | 40  (14.1%) | 47  (19.9%) | 33  (14.4%) |
| ≥ 6.0mm,  < 7.0mm | 13 (6.0%) | 44  (16.9%) | 2  (2.4%) | 0  (0%) | 2  (1.4%) | 45  (15.8%) | 30  (12.7%) | 11  (4.8%) |
| ≥ 7.0mm,  < 8.0mm | 2  (0.9%) | 27  (10.3%) | 0  (0%) | 0  (0%) | 4  (2.8%) | 66  (23.2%) | 13  (5.5%) | 10  (4.4%) |
| ≥ 8.0mm,  < 9.0mm | 0  (0%) | 5  (1.9%) | 0  (0%) | 0  (0%) | 0  (0%) | 44  (15.5%) | 6  (2.5%) | 0  (0%) |
| ≥ 9.0mm,  < 10.0mm | 0  (0%) | 5  (1.9%) | 0  (0%) | 0  (0%) | 1  (0.7%) | 30  (10.5%) | 1  (0.4%) | 0  (0%) |
| ≥ 10.0mm,  < 11.0mm | 0  (0%) | 2  (0.8%) | 0  (0%) | 0  (0%) | 0  (0%) | 17  (6.0%) | 2  (0.8%) | 0  (0%) |
| ≥ 11.0mm | 0  (0%) | 0  (0%) | 0  (0%) | 0  (0%) | 0  (0%) | 15  (5.3%) | 1  (0.4%) | 0  (0%) |

* No patient had facial nodes

| Nodal groups | SAD |
| --- | --- |
|  | ICC*  (95%CI) |
| Parotid | 0.91  (0.85 – 0.95) |
| Submandibular | 0.82  (0.68 – 0.90) |
| Occipital | 0.89  (0.80 – 0.94) |
| Retropharyngeal | 0.83  (0.71 – 0.91) |
| Upper internal jugular |  |
| Jugulodigastric | 0.91  (0.84 – 0.95) |
| Level IIa | 0.89  (0.80 – 0.94) |
| Level IIb | 0.85  (0.74 – 0.92) |

Supplementary Table 2. Inter-observer agreement for SAD of the largest nodes*

*Inter-observer agreements for SAD for facial and retroauricular groups were not calculated as only 0 and 2 nodes were identified respectively in 50 randomly selected patients.

SAD = short axis diameter, ICC = Intraclass coefficient, CI = confidence interval


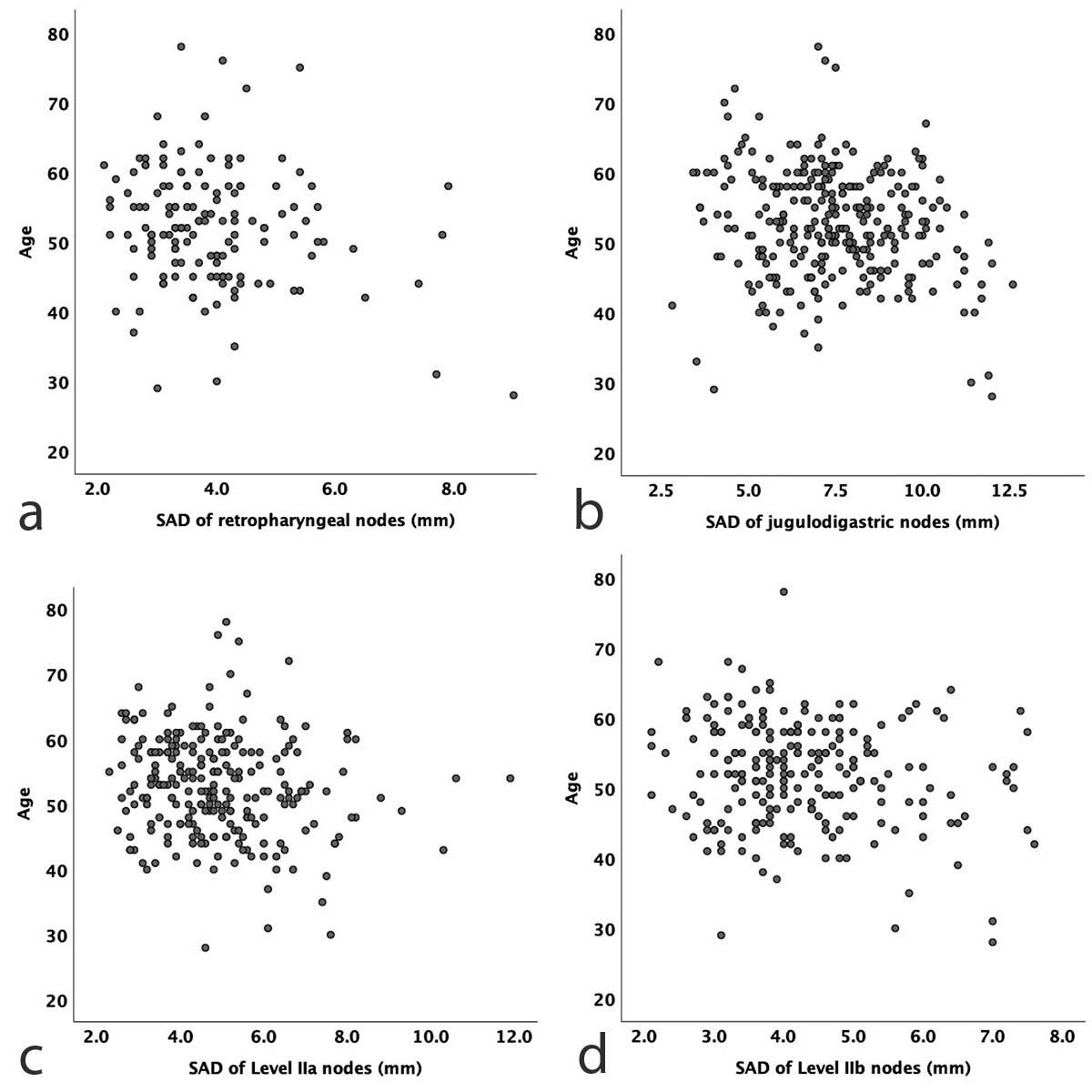


Supplementary Figure 1. Scatter plots show the negative correlation of age with the short axis diameter of the largest node in retropharyngeal (a), jugulodigastric (b), Level IIa (c) and Level IIb (d) nodes. The Pearson correlation coefficients are -0.19, -0.18, -0.15, and -0.16 for retropharyngeal, jugulodigastric, Level IIa and Level IIb nodes, respectively.
